# Association between preoperative anemia and postoperative short-term outcomes in patients undergoing colorectal cancer surgery - a propensity score matched retrospective cohort study

**DOI:** 10.1186/s12871-023-02270-2

**Published:** 2023-09-11

**Authors:** Ting Yan, Shaohui Lei, Bingbing Zhou, Yaqi Huang, Xiaoting Li, Jiaqi Zhang, Qijian Huang, Liangcheng Zhang

**Affiliations:** 1 Department of Anesthesiology, Fujian Medical University Union Hospital, No. 29 Xin-Quan Road, Fuzhou, 350001 China; 2 Department of Anesthesiology, Nanfang Hospital, Southern Medical University, Guangzhou, China; 3 Department of Information Systems and Management Engineering, Southern Science and Technology University, Shenzhen, China

**Keywords:** Preoperative anemia, Colorectal cancer surgery, Major morbidity, Propensity score matching

## Abstract

**Background:**

Based on previous studies which failed to analyze important confounding variables, the association between preoperative anemia and outcomes of patients who underwent colorectal cancer (CRC) surgery has not been clearly demonstrated. This study aimed to investigate the relationship between preoperative anemia and short-term outcomes in patients with CRC.

**Methods:**

Data from a retrospective collective database of patients who underwent CRC surgery at our hospital between September 1, 2019 and September 30, 2021 were retrieved and analyzed, and the short-term postoperative outcomes of anemic (hemoglobin < 120 g dL^− 1^ for female, hemoglobin < 130 g dL^− 1^ for male) and non-anemic patients were analyzed, using a 1:1 propensity score matching (PSM) analysis.

**Results:**

After excluding some cases, the remaining 1894 patients had complete data available for analysis. The incidence of preoperative anemia was 39.8% (754/1894). Before PSM, preoperative anemia patients had a higher risk of major morbidity than non-anemia patients (27.2% vs. 23.1%, odds ratio [OR] 1.245, 95% confidence interval [CI] 1.008–1.538, *P* = 0.042). After PSM was performed in the cohort, 609 patients remained in the anemic and non-anemic groups. The incidence of major morbidity (25.8% vs. 24.0%, OR 1.102, 95% CI 0.849–1.429, *P* = 0.446) between anemic and non-anemic patients was comparable. No significant difference was found between the anemic and non-anemic groups in postoperative length of stay (8.0 [6.0–12.0] vs. 8.0 [7.0–11.0], *P* = 0.311). The sensitivity analysis results were in accordance with the primary outcome. Furthermore, we did not ascertain any discernible correlation between the extent of anemia and significant major morbidity.

**Conclusions:**

Compared with preoperative non-anemia, anemia status does not seem to be associated with major morbidity in patients with CRC surgery. It is noteworthy that, anemia is insufficient as a solitary risk factor and may be a better marker of poor health resulting from multiple factors.

**Trial registration:**

Registration Authority: Chinese Clinical Trial Registry; Registration number and date: ChiCTR2100049696, 08/08/2021; Principal investigator: Ting Yan; Link to trial registry: http://www.chictr.org.cn/showproj.aspx?proj=131698; .

**Supplementary Information:**

The online version contains supplementary material available at 10.1186/s12871-023-02270-2.

## Introduction

Colorectal cancer (CRC) is the fourth most common malignancy worldwide [1]. Evidence suggests that more than 95% patients with CRC benefit from surgical treatment [2]. Despite the improved safety in CRC surgery, complications, including intestinal obstruction, anastomotic fistula, peritonitis, and bleeding, remain the leading cause of postoperative morbidity and are reported at approximately 5–15% [3, 4].

Anemia is a common preoperative morbidity, with an incidence of approximately 15–26% [5, 6]. A study involving 227,425 patients reported by Musallam and colleagues [7] demonstrated that preoperative anemia was a risk factor for the 30-day postoperative mortality and incidence of composite complications. However, for approximately 7% of patients in that study [7], the preoperative hematocrit level was obtained > 4 weeks before surgery and probably was not reflective of the exact concentration of red blood cells at the time of surgery. Furthermore, the article did not show perioperative blood transfusion events in the patients, although perioperative blood transfusion is known to be associated with increased morbidity and mortality in surgical patients [8]. Jiang et al. [9] established a prediction model in 2019 for predicting postoperative intestinal complications after colorectal surgery and indicated preoperative anemia as risk factor for postoperative intestinal complications. However, another report by Bruns and colleagues [10] in the same year had conflicting results. The causes of the different results in these two studies have not been well demonstrated. Additionally, neither Bruns [10] nor Jiang [9] considered blood transfusions into account. Thus, we believe that previous studies [7, 9, 10] have limitations and do not clearly clarify the relationship between anemia and outcomes. In addition, Kamonvarapitak and colleagues [11] found that lymphocyte-to-monocyte ratio (LMR) can serve as a predictor of postoperative infectious complications after laparoscopic CRC surgery.

To clarify this clinical uncertainty, we conducted a retrospective study covering the variables of suspected confounding factors, especially perioperative blood transfusion and LMR, to evaluate the relationship between preoperative anemia and short-term postoperative outcomes after CRC surgery. We hypothesized that preoperative anemia would increase the risk of major morbidity (including surgical site infection, anastomotic bleeding, anastomotic leakage, chylous ascites, bloodstream infection, myocardial infarction, congestive heart failure, stroke/transient ischemic attack, pulmonary embolism, and pneumonia). The primary outcome was the incidence of major morbidity, while the secondary outcomes were the incidence ofthe major components of major morbidity (including surgical site infection, anastomotic bleeding, anastomotic leakage, and chylous ascites, pneumonia) and postoperative length of stay. This study aimed to investigate the association between preoperative anemia and major morbidity in patients with CRC surgery, and ultimately, the goal was achieved.

## Materials and methods

### Study design and population

This study was designed to collect perioperative data from patients undergoing CRC surgery at the Fujian Medical University Union Hospital. This study was approved by the Ethics Committee of Fujian Medical University Union Hospital (approval number: 2021KY058; approval date: May 8, 2021). The need for written informed consent was waived by the Fujian Medical University Union Hospital Ethics Committee due to retrospective nature of the study. The methods were performed in accordance with relevant guidelines and regulations. The trial was registered prior to patient enrollment in the Chinese Clinical Trial Registry (ChiCTR2100049696, 08/08/2021Ting Yan,http://www.chictr.org.cn/showproj.aspx?proj=131698, ). We have registered our protocol in the Chinese Clinical Trail Registry (ChiCTR2100049696). This study adhered to the applicable Strengthening the Reporting of Observational studies in Epidemiology (STROBE) guidelines, and the checklist is shown in Supplementary material–Table S7. To detect the association between preoperative anemia and postoperative complications during the hospitalization period, all identified patients were divided into two cohorts according to the presence or absence of preoperative anemia, and the confounding variables between the two groups were balanced using PSM. All related clinical data for this retrospective study were collected from the scientific research database and the electronic medical record system of the Union Hospital of Fujian Medical University. We started collecting data on November 1, 2021 and end the collection on March 1, 2022. Although we could access to information that could identify individual participants during or after data collection, we keep patients’ information strictly confidential. The diagnosis of colonic or rectal carcinoma in each case included in the study was confirmed based on histopathological characteristics. The inclusion criteria for this study were patients aged 18 years or older who underwent CRC selective surgery during September 1, 2019 and September 30, 2021. The following patients were excluded: benign tumors, nonradical surgery, preoperative admission to the intensive care unit (ICU), lack of clinical data (including preoperative hemoglobin levels), and undetermined tumor node metastasis (TNM) stages.

### Study outcomes and variables definition

The primary outcome was the incidence of major morbidity, which was defined as the presence of at least one of the following conditions: surgical site infection (superficial/deep incisional, organ, or space) [12], anastomotic bleeding [13], anastomotic leakage [14], chylous ascites [15], bloodstream infection (sepsis/bacteremia) [12], myocardial infarction [16], congestive heart failure [16], stroke/transient ischemic attack [17], pulmonary embolism [16], and pneumonia [18]. Secondary outcomes include the incidence of surgical site infection, anastomotic bleeding, anastomotic leakage, chylous ascites, pneumonia, and postoperative length of stay). Observational measures were not included in the statistical analysis, including postoperative death, unplanned secondary surgery, and components with a lower incidence of major morbidity. All postoperative complications were observed during hospitalization.

We investigated the patients’ baseline characteristics and perioperative management. The CRC surgeries included left colectomy, right colectomy, subtotal colectomy, total colectomy, transverse colectomy, low anterior resection, and abdominoperineal resection. ≤ ≤ Hemoglobin (Hb) levels below 120 g L^− 1^ in women and 130 g L^− 1^ in men were identified as indicators of anemia. Anemia was further categorized into three levels for both genders: mild (Hb ≥ 90 g L^− 1^), moderate (60 g L^− 1^ ≤ Hb < 90 g L^− 1^), and severe (Hb < 60 g L^− 1^) [19]. We ascertained patients’ hemoglobin levels within a 7-day timeframe, specifically in close proximity to the commencement of surgery, as part of our preoperative assessment. Cardiovascular comorbidities included hypertension, ischemic heart disease, atrial fibrillation, pulmonary hypertension, defibrillator implantation, prior valve repair, pacemaker implantation, heart failure, cardiomyopathy, prior percutaneous coronary intervention, and prior coronary artery bypass grafting. Pulmonary comorbidities included asthma, chronic obstructive pulmonary disease, bronchiectasis, and emphysema. Perioperative blood transfusions include preoperative, intraoperative, and postoperative blood transfusions. In perioperative blood management, surgeons and anesthesiologists follow a restrictive transfusion strategy [20] at our institution.

### Sample size calculation

The incidence of major morbidity in the matched non-anemia group was 24.0%. A total of 1218 cases with an incidence of 24% in the composite outcome provided 85% power to detect an odds ratio [OR] of 1.5 or higher in anemic patients and non-anemic patients. During the establishment of a logistic regression model, we followed the at least “ten events per variable” rule [21] to ensure sufficient accuracy of regression.

### Statistical analysis

To minimize the influence of confounding factors and potential bias between the anemic groups and the non-anemic group before surgery, the propensity score was calculated using logistic regression, and the 1:1 matching method was used to compare the incidence of postoperative morbidity between the anemic group and the non-anemic groups. Based on the estimated propensity score for each patient, we used nearest neighbor matching with a caliper distance of 0.2 [22, 23]. Standardized mean difference (SMD) was used to assess intergroup equilibrium before and after matching of the study population. Baseline variables with an SMD≤$1.96\times \sqrt{(n1+n2)?(n1\times n2)}$ were considered balanced between groups [24]. In performing PSM we adjusted for covariates associated with anemia obtained by multivariate logistic regression.

Sensitivity analysis using multivariate logistic regression analysis of the entire cohort to identify independent risk factors for major morbidity. We also performed propensity score inverse probability of treatment weighting (IPTW) to validate the association between preoperative anemia status and major morbidity. We also analyzed clinical data in a cohort with or without anemia defined by hematocrit concentration [25] (less than 39% for male; less than 36% for female) using PSM to clarify the relationship between preoperative anemia and primary and secondary outcome. To explore potential correlations between anemia degree and postoperative complications, we devised another two PSM within the cohort: “Moderate Anemia” vs. “Mild Anemia + No Anemia” and “Moderate Anemia” vs. “No Anemia”. Furthermore, a subgroup analysis pertaining to “perioperative blood transfusions” was undertaken.

Continuous variables were expressed as medians (interquartile range), and categorical variables were expressed as frequencies and percentages. The Mann–Whitney U test was used for continuous variables, and the Pearson chi-square test or Fisher’s exact test was used for categorical variables to analyze differences between groups. All statistical analyses were performed using the SPSS software (version 22.0, IBM Corp., Armonk, NY, USA). PSM was performed using “PS Matching 3.04” and “SPSS Statistics R Essentials 22.0”. All *P*-values were based on bilateral statistical analysis, and *P* < 0.05 was considered statistically significant.

## Results

### Patient recruitment

**Fig. 1:**
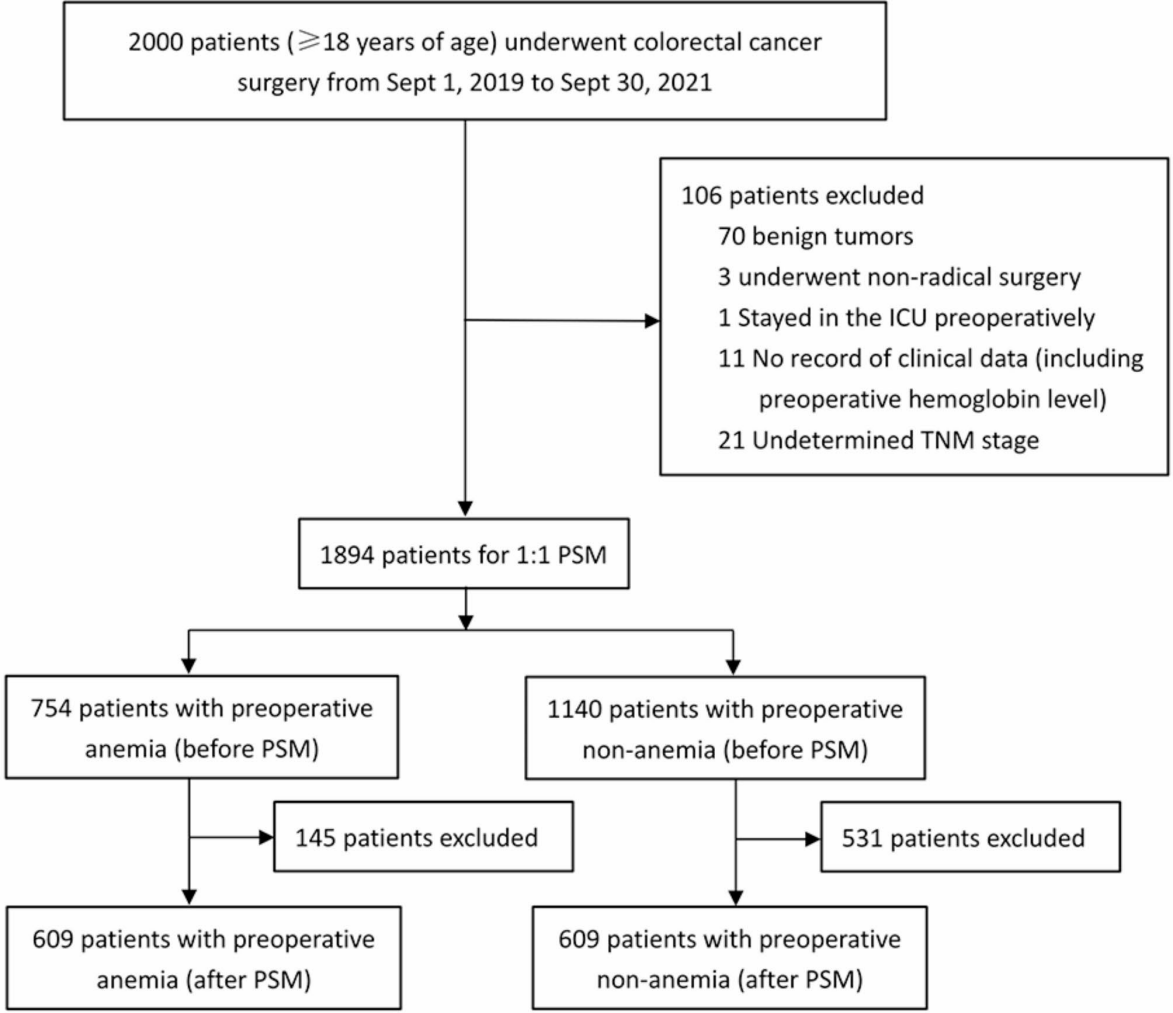
Flow chart of patients’ selection. PSM, propensity score matching

We identified 2000 patients aged 18 years or older who underwent CRC surgery between September 1, 2019 and September 30, 2021. After excluding 106 patients, a cohort of 1,894 patients with complete data remained to be matched. There were 754 patients (39.8%, 754/1894) with anemia and 1140 patients (60.2%, 1140/1894) without anemia before surgery. After a 1:1 propensity score match, 609 anemic patients and 609 nonanemic patients remained in our study, with 1218 cases eventually being analyzed. (Fig. 1)

### Hemoglobin values in anemic patients

According to the anemia criteria of different sexes, in the pre-matching anemia cohort, there were 629 (83.4%) patients with mild anemia and 125 (16.6%) patients of moderate anemia, and the mean preoperative hemoglobin was 111.0 [97.8, 119.0] in entire cohort. There were 629 /754 (83.4%) for mild anemia (hemoglobin: 112.8 ± 10.6 g L^− 1^; range from 91 to 129 g L^− 1^) and 125 / 754 (16.6%) and 125 /754 (16.6%) for moderate anemia (hemoglobin: 81.1 ± 5.99 g L^− 1^; range from 62 to 90 g L^− 1^). The hemoglobin of anemic group and non-anemic group was 107.6 ± 15.5 g L^− 1^ (range from 62 to 129 g L^− 1^) and 139.9 ± 11.5 (range from 120 to 180 g L^− 1^), respectively.

Perioperative blood transfusions events occurred 121 cases, including 37 preoperative, 23 intraoperative, and 68 postoperative blood transfusions. Among the 37 patients who received preoperative blood transfusions, five patients did not have their hemoglobin levels measured between the completion of the transfusions and the beginning of the surgery. Among the remaining 32 patients with preoperative blood transfusions, the lowest pretransfusion hemoglobin was 46 g L^− 1^, the highest pretransfusion hemoglobin was 86 g L^− 1^, the average pretransfusion hemoglobin was 63.1 ± 9.2 g L^− 1^, and the average blood transfusions volume was 3.5 ± 1.6 units. The average hemoglobin was 87.0 ± 11.6 g L^− 1^ after transfusions. In China, the volume of one unit of red blood cells approximately 200 mL.

### Identification of covariates associated with preoperative anemia

We performed multivariate logistic regression analysis and found that confounding variables associated with anemia included current smoking status, hypoalbuminemia, hypertension, preoperative chemoradiotherapy, tumor location, perioperative blood transfusions, age, body mass index (BMI), and LMR. These factors served as adjusted covariates in PSM. (Supplementary material–Table S2)

### Characteristics of patients before and after PSM

**Fig. 2:**
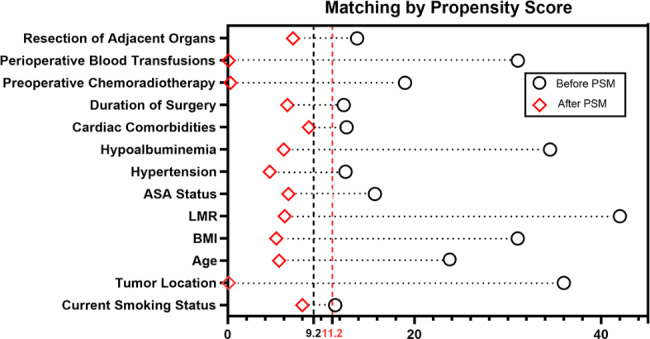
Illustration of Standardized Mean Differences in variables before and after PSM cohorts. ASA American society of anesthesiologists, LMR lymphocyte-to-monocyte ratio, BMI body mass index, PSM, propensity score matching, SMD standardized mean difference. The vertical dashed line in the Fig. 2 indicates the cut-off value of SMD for balance between groups before (black color) and after (red color) propensity score matching, respectively

Before matching, the SMD calculated according to the relevant formulas was less than 0.092, indicating a balance between the groups. We found that age, preoperative American Society of Anesthesiologists (ASA) physical status III–IV, preoperative cardiovascular and pulmonary comorbidity, TNM stage III–IV and intraoperative resection of adjacent organs in the anemia group were all higher than those in the non-anemia group, and the duration of surgery was longer in the anemia group. BMI, fraction of hypoalbuminemia, and LMR were lower in the anemic group than in the non-anemic group. In addition, the anemia group had a lower proportion of current smoking status and a higher proportion of preoperative chemoradiotherapy. After matching, all variables between the two groups reached equilibrium (SMD < 0.112). (Table 1; Fig. 2)

**Table 1:** Clinical variables between no anemia group and anemia group, before and after propensity score matching

Variables		Cohort before propensity matching			Cohort after propensity matching		
		No anemia n = 1140	Anemia n = 754	SMD ^*a*^	No anemia n = 609	Anemia n = 609	SMD ^*a*^
**ASA Status**	I–II	981 (86.1)	601 (79.7)	0.158	512 (84.1)	496 (81.4)	0.065
	III–IV	159 (13.9)	153 (20.3)		97 (15.9)	113 (18.6)	
**Gender**	Female	439 (38.5)	311 (41.2)	0.056	257 (42.2)	241 (39.6)	0.053
**Current Smoking Status** ** (within 12 months)**	Yes	266 (23.3)	142 (18.8)	0.115	142 (23.3)	123 (20.2)	0.080
**Cardiovascular Comorbidities** ^***b***^	Yes	60 (5.3)	67 (8.9)	0.127	35 (5.7)	50 (8.2)	0.087
**Pulmonary Comorbidities** ^***c***^	Yes	140 (12.3)	118 (15.6)	0.093	95 (15.6)	89 (14.6)	0.027
**History of Laparotomy**	Yes	178 (15.6)	130 (17.2)	0.043	109 (17.9)	109 (17.9)	< 0.001
**Hypoalbuminemia**	< 3.5 g L^− 1^	12 (1.1)	94 (12.5)	0.345	12 (2.0)	24 (3.9)	0.060
**Hypertension**	Yes	329 (28.9)	263 (34.9)	0.126	194 (31.9)	207 (34.0)	0.045
**Diabetes**	Yes	157 (13.8)	119 (15.8)	0.055	77 (12.6)	94 (15.4)	0.077
**TNM stage** ^***d***^	0–II	664 (58.2)	404 (53.6)	0.093	347 (57.0)	326 (53.5)	0.069
	III–IV	476 (41.8)	350 (46.2)		262 (43.0)	283 (46.5)	
**Preoperative Chemoradiotherapy**	Yes	276 (24.3)	250 (33.2)	0.190	212 (34.8)	211 (34.6)	0.003
**Tumor Location**	Rectum	682 (59.8)	317 (42.0)	0.360	293 (48.1)	293 (48.1)	< 0.001
	Colon	458 (40.2)	437 (58.0)		316 (51.9)	316 (51.9)	
**Age**	Years	60.0 [52.0, 68.0]	64.0 [55.0, 72.0]	0.240	62.0 [54.0, 69.0]	63.0 [54.0, 71.0]	0.055
**BMI**	Kg m-^2^	23.4 [21.5, 25.7]	22.3 [20.4, 24.6]	0.311	22.8 [20.8, 24.8]	22.5 [20.7, 24.8]	0.052
**LMR**		4.7 [3.3, 6.1]	3.6 [2.4, 4.9]	0.420	4.1 [2.8, 5.4]	3.8 [2.6, 5.2]	0.061
**Duration of surgery**	Min	210.0 [175.0, 254.0]	220.0 [184.0, 269.0]	0.124	215.0 [175.0, 260.0]	220.0 [182.0, 268.0]	0.064
**Surgical Approach**	Laparoscopic	1096 (96.1)	717 (95.1)	0.048	578 (94.9)	582 (95.6)	0.030
	Open	44 (3.9)	37 (4.9)		31 (5.1)	27 (4.4)	
**Robot-assisted Surgery**	Yes	129 (11.3)	67 (8.9)	0.085	67 (11.0)	62 (10.2)	0.029
**Preventive Stoma**	Yes	320 (28.1)	167 (22.1)	0.143	170 (27.9)	153 (25.1)	0.067
**Resection of Adjacent Organs**	Yes	71 (6.2)	79 (10.5)	0.139	44 (7.2)	57 (9.4)	0.070
**Intraperitoneal Chemotherapy**	Yes	25 (2.2)	13 (1.7)	0.036	14 (2.3)	10 (1.6)	0.050
**Perioperative Blood Transfusions**	Yes	26 (2.3)	95 (12.6)	0.311	24 (3.9)	24 (3.9)	< 0.001

Data are presented as n (%) or median [IQR]

SMD standardized mean difference, ASA American society of anesthesiologists, BMI body mass index, LMR lymphocyte-to-monocyte ratio, TNM tumor node metastasis

^*a*^ SMD ≤ 0.092 and 0.112were considered to be balanced between the two groups before and after matching, respectively^21^

^*b*^ Cardiovascular Comorbidity includes ischemic heart disease, automated implantable cardioverter defibrillators insertion, cardiac valve replacement, pacemaker insertion, cardiomyopathy, percutaneous coronary intervention, coronary artery bypass graft, atrial fibrillation

^*c*^ Pulmonary Comorbidity includes asthma, chronic obstructive pulmonary disease, bronchiectasis, emphysema

^*d*^ TNF 0 stage that pathologic complete response (ypCR)

### Outcomes before and after PSM

In the pre-matching cohort, patients with anemia had an increased risk of major morbidity compared to patients without preoperative anemia (27.2% vs. 23.1%, OR 1.245, 95% confidence interval [CI] 1.008–1.538, *P* = 0.042). Among the secondary outcomes, postoperative death (1/1140 vs. 3/754) and unplanned secondary surgery (7/1140 vs. 6/754) lost the possibility of group comparison because of their very low incidence. There was no significant difference in the incidence of major surgical complications between the two groups (OR 1.119, 95% CI 0.883–1.418, *P* = 0.352). However, the postoperative length of stay in the anemia group was longer than in the non-anemia group (8.0 [7.0, 13.0] vs. 8.0 [6.0, 11.0], *P* < 0.001). (Table 2)

**Table 2:** Postoperative outcomes in cohort before propensity matching (n = 1894)

Characteristics		No anemia n = 1140	Anemia n = 754	OR	95%CI	*P*-value ^*d*^
**Primary Outcome**	Major Morbidity ^*a*^	263 (23.1)	205 (27.2)	1.245	1.008–1.538	0.042
**Secondary Outcome**	Surgical Site Infection ^*b*^	117 (10.3)	91 (12.1)	1.200	0.897–1.605	0.124
	Anastomotic Bleeding	22 (1.9)	13 (1.7)	0.892	0.446–1.781	0.862
	Anastomotic Leakage	53 (4.6)	41 (5.4)	1.179	0.776–1.792	0.451
	Chylous Ascites	45 (3.9)	26 (3.4)	0.869	0.521–1.421	0.622
	Pneumonia	100 (8.8)	102 (13.5)	1.627	1.214–2.180	0.001
	Postoperative Length of Stay	8.0 [6.0, 11.0]	8.0 [7.0,13.0]	-	-	< 0.001
	Bloodstream Infection ^*c*^	5 (0.4)	7 (0.9)	-	-	-
	Myocardial Infarction	1 (0.1)	0 (0.0)	-	-	-
	Congestive Heart Failure	1 (0.1)	3 (0.4)	-	-	-
	Stroke/Transient Ischemic Attack	0 (0.0)	1 (0.1)	-	-	-
	Pulmonary Embolism	4 (0.4)	1 (0.1)	-	-	-
	Postoperative Death	1 (0.1)	3 (0.4)	-	-	-
	Unplanned Secondary Surgery	7 (0.6)	6 (0.8)	-	-	-

Data are presented as n (%) or median [IQR]

^*a*^ Major morbidity includes surgical site infection, anastomotic bleeding, anastomotic fistula, chylous ascites, bloodstream infection, myocardial infarction, congestive heart failure, stroke/transient ischemic attack, pulmonary embolism, and pneumonia

^*b*^ Surgical Site Infection include superficial/deep incisional, organ or space

^*c*^ Bloodstream Infection include sepsis and bacteremia

^*d*^ Chi-Square test was used for categorical data and Mann-Whitney U test was used for continuous data; Bonferroni correction is used in secondary outcome, P < 0.08 is considered to have statistically significant in the analysis of secondary outcome

The results showed that there was no statistical difference in the major morbidity between the anemia and non-anemia groups in the matched population (OR 1.102, 95% CI 0.849–1.429, *P* = 0.446). Similarly, in the secondary outcomes, major surgical complications (OR 1.082, 95% CI 0.806–1.453, *P* = 0.599) and postoperative length of stay (8.0 [6.0, 12.0] vs. 8.0 [7.0, 11.0], *P* = 0.311) were comparable between the anemic and non-anemic groups. The incidence rates of postoperative death and unplanned secondary surgery remain incomparable. (Table 3)

**Table 3:** Postoperative outcomes in cohort after propensity matching (n = 1218)

Characteristics		No anemia n = 609	Anemia n = 609	OR	95%CI	*P*-value ^*d*^
**Primary Outcome**	Major Morbidity ^*a*^	146 (24.0)	157 (25.8)	1.102	0.849–1.429	0.466
**Secondary Outcome**	Surgical Site Infection ^*b*^	60 (9.9)	70 (11.5)	1.126	0.785–1.614	0.291
	Anastomotic Bleeding	13 (2.1)	5 (0.8)	0.610	0.251–1.483	0.379
	Anastomotic Leakage	24 (3.9)	37 (6.1)	1.450	0.859–2.427	0.195
	Chylous Ascites	21 (3.4)	22 (3.6)	0.876	0.488–1.570	0.655
	Pneumonia	63 (10.3)	77 (12.6)	1.292	0.909–1.837	0.153
	Postoperative Length of Stay	8.0 [7.0, 11.0]	8.0 [6.0, 12.0]	-	-	0.311
	Bloodstream Infection ^*c*^	4 (0.7)	2 (0.3)	-	-	-
	Myocardial Infarction	1 (0.2)	0 (0.0)	-	-	-
	Congestive Heart Failure	0 (0.0)	2 (0.3)	-	-	-
	Stroke/Transient Ischemic Attack	0 (0.0)	1 (0.2)	-	-	-
	Pulmonary Embolism	4 (0.7)	0 (0.0)	-	-	-
	Postoperative Death	1 (0.2)	2 (0.3)	-	-	-
	Unplanned Secondary Surgery	4 (0.7)	4 (0.7)	-	-	-

Data are presented as n (%) or median [IQR]

^*a*^ Major morbidity includes surgical site infection, anastomotic bleeding, anastomotic fistula, chylous ascites, bloodstream infection, myocardial infarction, congestive heart failure, stroke/transient ischemic attack, pulmonary embolism, and pneumonia

^*b*^ Surgical Site Infection include superficial/deep incisional, organ or space

^*c*^ Bloodstream Infection include sepsis and bacteremia

^*d*^ Chi-Square test was used for categorical data and Mann-Whitney U test was used for continuous data; Bonferroni correction is used in secondary outcome, P < 0.08 is considered to have statistically significant in the analysis of secondary outcome

### Sensitivity analysis

Multivariable logistic regression analysis of the pre-matched cohort showed that male sex (OR 1.548), diabetes (OR 1.622), open surgery (OR 2.054), perioperative blood transfusions (OR 2.450), and longer duration of surgery (OR 1.004) were independent risk factors for major morbidity. However, preoperative anemia was not independently associated with major morbidity (OR 1.088, 95% CI 0.870–1.361, *P* = 0.460). (Table 4)

**Table 4:** Independent risk factors for major morbidity ^*a*^ in the entire cohort (n = 1894)

Variables	Univariate analysis ^*e*^			Multivariate analysis ^*f*^		
	OR	95%CI	*P*-value	OR	95%CI	*P*-value
**Preoperative Anemia**	1.245	1.008–1.538	0.042	1.088	0.870–1.361	0.460
**Sex** ^***b***^	1.542	1.235–1.920	< 0.001	1.548	1.233–1.943	< 0.001
**Diabetes**	1.587	1.204–2.093	0.001	1.622	1.222–2.154	0.001
**Surgical Approach** ^***c***^	2.299	1.459–3.620	< 0.001	2.054	1.276–3.309	0.003
**Duration of Surgery**	1.005	1.003–1.006	< 0.001	1.004	1.003–1.006	< 0.001
**Perioperative Blood Transfusions** ^***d***^	2.744	1.887–3.990	< 0.001	2.450	1.646–3.645	< 0.001

^*a*^ Major morbidity includes surgical site infection, hemorrhage, anastomotic fistula, chyle fistula, bloodstream infection,

myocardial infarction, congestive heart failure, stroke/transient ischemic attack, pulmonary embolism, and pneumonia

^*b*^ Woman as reference group

^*c*^ Laparoscopic group as reference group

^*d*^ Perioperative blood transfusion includes preoperative, intraoperative, and postoperative blood transfusion

^*e*^ Univariate logistic regression analysis

^*f*^ Multivariate logistic regression analysis

We did not find an association between preoperative anemia and major morbidity using the propensity score IPTW method (OR 1.035, 95% CI 0.881–1.204, *P* > 0.05). In the hematocrit concentration-defined anemia criterion, the incidence of major morbidity after PSM was comparable between the preoperative anemic and non-anemic groups (OR 1.105, 95% CI 0.848–1.440, *P* = 0.458). (Table 5)

**Table 5:** propensity score IPTW analysis was used to examine the association between anemia and major morbidity ^a^

	Multivariate analysis ^*e*^		
	OR	95%CI	*P*-value
**Preoperative Anemia**	1.099	0.947–1.278	0.215
**Sex** ^***b***^	1.528	1.327–1.760	< 0.001
**Diabetes**	1.778	1.473–2.142	< 0.001
**Surgical Approach** ^***c***^	1.863	1.345–2.564	< 0.001
**Duration of Surgery**	1.005	1.004–1.006	< 0.001
**Perioperative Blood Transfusions** ^***d***^	2.341	1.811–3.018	< 0.001

^*a*^ Major morbidity includes surgical site infection, hemorrhage, anastomotic fistula, chyle fistula, bloodstream infection,

myocardial infarction, congestive heart failure, stroke/transient ischemic attack, pulmonary embolism, and pneumonia

^*b*^ Woman as reference group

^*c*^ Laparoscopic group as reference group

^*d*^ Perioperative blood transfusion includes preoperative, intraoperative, and postoperative blood transfusion

^*e*^ Multivariate logistic regression analysis

These negative results were in line with previous PSM analyses, indicating the robustness of our study.

Eventually, no association between the degree of anemia and short-term postoperative complications (Supplementary material–Tables S3 and S4) and Clavien-Dindo classification (Supplementary material–Table S6) in CRC patients remains found in our study. Among the population undergoing perioperative blood transfusions, it is noteworthy that patients with anemia demonstrated a surprising occurrence of reduced rates in major morbidity (with an odds ratio of 0.188, as indicated in Supplementary material–Table S5).

## Discussion

In this single-center retrospective cohort study, we adjusted for suspected confounders by PSM but did not find a relationship between preoperative anemia and major morbidity. The sensitivity analyses demonstrated the reliability of the results of this study.

Preoperative anemia has been shown to increase morbidity and mortality in patients [26, 27], and mild anemia is an independent risk factor for poor postoperative outcomes [28]. Anemia impairs oxygen supply, resulting in delayed wound healing, reduced muscle performance, and increased fatigue [28, 29]. An epidemiological survey [30] showed that approximately one-third of patients undergoing elective major surgery had preoperative anemia. The potential risk and high incidence of preoperative anemia demonstrate the importance of this issue and the immense potential for improvement in perioperative management.

In this study, we did not observe a relationship between preoperative anemia and major postoperative morbidity, which is in line with the studies conducted by Hardy [31] and Bruns [10]. However, previous studies have reported an association between preoperative anemia and postoperative complications [27, 32, 33], which contradicts our findings and may be due to inconsistencies in the types of surgical procedures. Different surgeries could cause different degrees of stress and inflammation in the body. The impact of anemia status on patients undergoing different procedures requires further investigation. Based on available evidence, our findings are limited to patients undergoing CRC surgery.

A meta-analysis [34] revealed that correcting anemia before surgery significantly lowered the risk of perioperative blood transfusion in patients undergoing arthroplasty, showing a relative risk of 0.48. This evidence opens the possibility of whether correction of preoperative anemia improves patient outcomes. Presently, the evidence supporting the aggressive management of preoperative anemia to reduce perioperative morbidity and mortality is limited. Nevertheless, some significant randomized controlled trials [35, 36] are currently underway to shed more light on this matter. However, it is important to note that no substantial, high-quality trials have been published yet, directly comparing preoperative anemia treatment with standard treatment or placebo. Consequently, we must rely on indirect evidence at this stage. Numerous studies have indicated that administering iron therapy to treat iron deficiency anemia leads to increased hemoglobin levels and reduced red blood cell transfusions [36]. Nonetheless, the impact of this treatment on outcomes for other patients requires further investigation.

In the multivariate analysis for major morbidity, the identified risk factors (including sex, duration of surgery, and perioperative blood transfusions) were consistent with those in previous studies [37], but anemia is not included. This result [37] just similar to our study, supported that perioperative blood transfusions rather than preoperative anemia was associated with major morbidity. We found that patients with anemia were older, had higher ASA status scores and more preoperative comorbidities, which was similar to Bruns’ observation [10]. However, whether anemia acts as a causal factor or a confounding symptom reflecting poor physical status and disease invasion remains controversial [10, 38, 39]. In our opinion, anemia may be indicative of overall poor health.

One issue deserves to be raised: as a composite endpoint, the major morbidity in our study has its own limitations. Composite endpoints may have different results under different definitions [10, 27, 31], although it is easier to obtain a sufficient sample size with statistical efficacy and a shorter study period for a very low incidence of complications, such as myocardial infarction, and pulmonary embolism. Hence, additional analyses of the individual components of composite endpoints are also needed [40]. Our primary a composite of ten complications, respectively, and we did not reanalyze all specific complications because of the low rate of complications. We investigated complications that had a significant impact on patients, rather than all those that had a different degree impact on patients. Subsequent large-population studies may provide an avenue for analyzing specific complications associated with major morbidity.

It is intriguing to note that within the group of patients receiving perioperative blood transfusions, those with anemia displayed surprisingly reduced occurrences of significant morbidity, as indicated by an odds ratio of 0.188. It’s worth noting that the subgroup analysis was conducted primarily for exploratory reasons, and given the relatively small size of the sample involving perioperative blood transfusion instances in this study, there is a clear need for additional research to comprehensively understand the intricate relationship between preoperative anemia and major morbidity influenced by perioperative blood transfusion.

The strengths of this study as follows: First, we investigated some important intraoperative variables, such as perioperative blood transfusions, adjacent organ resection, and LMR, which were not analyzed in previous studies [7, 32]. Secondly, we performed post-hoc sensitivity analyses, including IPTW, different definition of anemia and multivariate analysis, verifying the reliability of the primary outcome of this study.

Our study had the following limitations. First, although we were able to collect the preoperative serum creatinine values of the subjects, serum creatinine was not measured in all patients after surgery; therefore, the influence of anemia on postoperative acute kidney injury could not be evaluated. Second, the period of collection of complications in this study was short-term during postoperative hospitalization in the last 2 years; therefore, data on long-term mortality could not be obtained nor investigated. Third, owning to the nature of the study, we could not avoid the limitations of single-center and retrospective studies. Further studies with a prospective study design and inclusion of patient-centered factors, such as the frailty index, will improve our understanding of the influence of preoperative anemia on patients postoperatively, as retrospective studies cannot collect patient-centered parameters in frailty index scales, such as weight loss, grip strength decline and fatigue [27]. Finally, the study was limited to only a single preoperative hemoglobin value before surgery and failed to distinguish between acute anemia and chronic anemia, thereby ignoring the dynamic change in hemoglobin.

## Conclusions

In this single-center retrospective study, we investigated the relationship between preoperative anemia and postoperative complications in patients with CRC using PSM. Significant differences in major morbidity between anemic and non-anemic groups were not found. Anemia has relatively limited clinical evidence associated with major postoperative morbidity in patients undergoing CRC surgery and is more appropriate as a warning sign.

### Electronic supplementary material

Below is the link to the electronic supplementary material.

Supplementary Material 1

## Data Availability

The datasets used and/or analysed during the current study are available from the corresponding author on reasonable request.

## Abbreviations

CRC: colorectal cancer
ICU: intensive care unit
ASA: American society of anesthesiologists
LMR: lymphocyte to monocyte ratio
WHO: world health organization
PCI: prior percutaneous coronary intervention
COPD: chronic obstructive pulmonary disease
SMD: standardized mean difference
PSM: propensity score matching
TNM: tumor node metastasis
BMI: body mass index
IPTW: inverse probability of treatment weighting
