# Supplementary material for: Association between preoperative anemia and postoperative short-term outcomes in patients undergoing colorectal cancer surgery - a propensity score matched retrospective cohort study
Source: BMC Anesthesiol. 2023 Sep 11;23:307. doi: 10.1186/s12871-023-02270-2 (PMC10494383; doi:10.1186/s12871-023-02270-2)
Supplement: Supplementary file 1 — Supplementary Material 1 [file 12871_2023_2270_MOESM1_ESM.docx]

Table S1 Diagnostic criteria for each component of the primary morbidity

| Complication | Diagnostic Criteria | Reference |
| --- | --- | --- |
| surgical site infection (SSI) | Including superficial incisional, deep incisional and organ/space SSI. Due to the large content, see pages 313 and 314 of the reference. | Am J Infect Control.  2008Jun;36(5):309-32. |
| anastomotic bleeding | One of the following scenarios to be postoperative anastomotic bleeding: patients who suffered from (a) postoperative blood stool; (b) hemostatic instability; or (c) hypovolemic shock that required either blood transfusions or other emergency interventions | Surg Laparosc Endosc Percutan Tech. 2014 Oct;24(5):465-9. |
| anastomotic fistula/ anastomosis leak | One of the following: postoperative peritonitis found at reoperation, fecaloid drain, fecal material from the wound, extravasation of contrast on enema, or the presence of air or fluid in the anastomotic region visualized by CT scan. Anastomotic leaks were classified as “major” (need of reoperation or percutaneous radiologic drainage, Clavien-Dindo grades III) and “minor” (conservative medical treatment, Clavien-Dindo grades I and II) | Int J Colorectal Dis . 2013 Jul;28(7):967-71.  Ann Surg. 2004 Aug;240(2):205-13. |
| chylous ascites | Abdominal drainage fluid is milky white, drainage volume is greater than 200 ml/d, and positive ascites chyle test can confirm the diagnosis. | J Urol. 2002 Feb;167(2 Pt 1):449-57. |
| bloodstream infection | Laboratory-confirmed bloodstream infection: it must meet at least 1 of the following criteria: 1. Patient has a recognized pathogen cultured from 1 or more blood cultures and organism cultured from blood is not related to an infection at another site. 2. Patient has at least 1 of the following signs or symptoms: fever (>38℃), chills, or hypotension and signs and symptoms and positive laboratory results are not related to an infection at another site and common skin contaminant is cultured from 2 or more blood cultures drawn on separate occasions. | Am J Infect Control.  2008Jun;36(5):309-32. |
| myocardial infarction | Acute myocardial injury with clinical evidence of acute myocardial ischaemia and with detection of an increase or decrease in cTn values with at least one value above the 99th percentile URL and at least one of the following: (i) Symptoms of myocardial ischaemia (ii) New ischaemic ECG changes (iii) Development of pathological Q waves (iv) Imaging evidence of new loss of viable myocardium or new regional wall motion abnormality in a pattern consistent with an ischaemic aetiology (v) Identification of a coronary thrombus by angiography or autopsy. | Br J Anaesth. 2021 Jan;126(1):56-66. |
| congestive heart failure | Requires at least one of the following clinical signs: (i) An elevated jugular venous pressure (ii) Respiratory rales/crackles and crepitations (iii) Presence of S3 and at least one of the following radiographic findings: (a) Vascular redistribution (b) Interstitial pulmonary oedema (c) Frank alveolar pulmonary oedema | Br J Anaesth. 2021 Jan;126(1):56-66. |
| stroke/transient ischemic attack | A. Sudden onset of fully reversible neurological or retinal symptoms (typically hemiparesis, hemihypesthesia, aphasia, neglect, amaurosis fugax, hemianopsia, or hemiataxia)  B. Duration <24 h  C (Modified). At least 2 of the following:  (i) All symptoms are maximal in <1 min (no gradual spread)  (ii) All symptoms occur simultaneously  (iii) All symptoms are deficits (no irritative symptoms such as photopsias, pins, and needles, etc)  (iv) No headache accompanies or follows the neurological symptoms within 1 h | N Engl J Med. 2020;382(20):1933-1941  Stroke. 2019;50(8):2080-2085. |
| pulmonary embolism | Diagnosis of pulmonary embolism requires any one of the following: (i) A high probability ventilation/ perfusion lung scan; (ii) An intraluminal filling defect of segmental or larger artery on a helical CT scan; (iii) An intraluminal filling defect on pulmonary angiography; (iv) A positive diagnostic test for deep venous thrombosis (e.g. positive compression ultrasound) and one of the following: (a) Non-diagnostic (i.e. low or intermediate probability) ventilation/perfusion lung scan (b) Non-diagnostic (i.e. sub-segmental defects or technically inadequate study) helical CT scan. | Br J Anaesth. 2021 Jan;126(1):56-66. |
| pneumonia | Postoperative pneumonia was considered clinically present when new and/or progressive pulmonary infiltrates were identified on chest radiographs or computed tomography in conjunction with two or more of the following criteria: fever (>38℃) without other identifiable causes, leukocytosis >12×109 /L) or leukopenia (<4×109 /L), and purulent secretions. The microbiological etiology of postoperative pneumonia was mainly identified by semiquantitative cultures from endotracheal aspiration of lower respiratory tract secretions or sputum with an initial microscopic examination combined with quantitative bacterial culture. | Clin Infect Dis 2016;63:e61-e111. |

Table S2 Covariates associated with the anemic in the entire cohort (n=1894)

| Variables | Multivariate logistic regression analysis | | |
| --- | --- | --- | --- |
|  | OR | 95%CI | *P*-value |
| Tumor location | 2.571 | 2.048-3.227 | <0.001 |
| Current Smoking Status | 0.706 | 0.548-0.910 | 0.007 |
| Preoperative chemoradiotherapy | 2.068 | 1.584-2.699 | <0.001 |
| Hypertension | 1.328 | 1.044-1.689 | 0.021 |
| Hypoalbuminemia | 8.306 | 4.317-15.979 | <0.001 |
| Perioperative blood transfusion | 4.574 | 2.803-7.463 | <0.001 |
| BMI | 0.895 | 0.865-0.926 | <0.001 |
| Age | 1.018 | 1.009-1.027 | <0.001 |
| LMR | 0.842 | 0.796-0.891 | <0.001 |

BMI body mass index, LMR lymphocyte-to-monocyte ratio.

This analysis was only to find covariates associated with anemic and non-anemic patients and then to be adjusted in propensity score matching, not to find risk factors for preoperative anemia.

Table S3 Comparison of postoperative complications between moderate and no moderate anemic groups after PSM (n=328)

| Outcome | | No moderate anemia (n=231) | Moderate anemia (n=97) | OR | 95%CI | *P*-value ^c^ |
| --- | --- | --- | --- | --- | --- | --- |
|  |  |  |  |  |  |  |
| Primary Outcome | Major Morbidity ^a^ | 69 (29.2) | 28 (30.4) | 1.059 | 0.626-1.790 | 0.831 |
| Secondary Outcome | Surgical Site Infection ^b^ | 84 (29.0) | 13 (34.2) | 1.275 | 0.623-2.611 | 0.505 |
|  | Anastomotic Bleeding | 95 (30.0) | 2 (18.2) | 0.519 | 0.110-2.449 | 0.613 |
|  | Anastomotic Leakage | 93 (29.4) | 4 (33.3) | 1.199 | 0.352-4.079 | 1.000 |
|  | Chylous Ascites | 93 (29.3) | 4 (36.4) | 1.376 | 0.394-4.814 | 0.868 |
|  | Pneumonia | 81 (29.5) | 16 (30.2) | 1.036 | 0.545-1.967 | 0.915 |
|  | Postoperative Length of Stay | 8.0 [7.0, 12.0] | 8.0 [7.0, 13.0] | - | - | 0.464 |
|  |  |  |  |  |  |  |

Data are presented as n (%) or median [IQR].
^a^ Major morbidity includes surgical site infection, anastomotic bleeding, anastomotic fistula, chylous ascites, bloodstream infection, myocardial infarction, congestive heart failure, stroke/transient ischemic attack, pulmonary embolism, and pneumonia.
^b^ Surgical Site Infection include superficial/deep incisional, organ or space.
^c^ Chi-Square test was used for categorical data and Mann-Whitney U test was used for continuous data; Bonferroni correction is used in secondary outcome, P<0.008 is considered to have statistically significant in the analysis of secondary outcome.

Table S4 Comparison of postoperative complications between moderate and no anemic groups after PSM (n=166)

| Outcome | | No anemia (n=92) | Moderate anemia (n=74) | OR | 95%CI | *P*-value ^c^ |
| --- | --- | --- | --- | --- | --- | --- |
|  |  |  |  |  |  |  |
| Primary Outcome | Major Morbidity ^a^ | 56 (46.3) | 18 (40.0) | 0.774 | 0.386-1.551 | 0.469 |
| Secondary Outcome | Surgical Site Infection ^b^ | 69 (45.7) | 5 (33.3) | 0.594 | 0.194-1.822 | 0.358 |
|  | Anastomotic Bleeding | 69 (43.1) | 5 (83.3) | 6.594 | 0.753-57.737 | 0.090 |
|  | Anastomotic Leakage | 72 (45.0) | 2 (33.3) | 0.611 | 0.109-3.432 | 0.693 |
|  | Chylous Ascites | 71 (44.9) | 3 (37.5) | 0.735 | 0.170-3.183 | 0.733 |
|  | Pneumonia | 66 (46.8) | 8 (32.0) | 0.535 | 0.217-1.319 | 0.170 |
|  | Postoperative Length of Stay | 8.5 [7.0, 12.0] | 8.0 [6.0, 10.0] | - | - | 0.113 |
|  |  |  |  |  |  |  |

Data are presented as n (%) or median [IQR].
^a^ Major morbidity includes surgical site infection, anastomotic bleeding, anastomotic fistula, chylous ascites, bloodstream infection, myocardial infarction, congestive heart failure, stroke/transient ischemic attack, pulmonary embolism, and pneumonia.
^b^ Surgical Site Infection include superficial/deep incisional, organ or space.
^c^ Chi-Square test was used for categorical data and Mann-Whitney U test was used for continuous data; Bonferroni correction is used in secondary outcome, P<0.008 is considered to have statistically significant in the analysis of secondary outcome.

Table S5 Subgroup-analysis of perioperative blood transfusions (relationship between preoperative anemia and major morbidity, n=1218)

|  | Perioperative blood transfusions cohort (n=48) | |  | No perioperative blood transfusions cohort (n=1170) | |
| --- | --- | --- | --- | --- | --- |
|  | OR (95%CI) | *P*-value |  | OR (95%CI) | *P*-value |
| Major Morbidity | 0.188 (0.052-0.674) | 0.008 |  | 1.211 (0.923-1.587) | 0.167 |
| Surgical Site Infection | 1.000 (0.248-4.028) | 1.000 |  | 1.205 (0.825-1.759) | 0.335 |
| Anastomotic Bleeding^*^ | - | - |  | 0.832 (0.252-2.741) | 0.762 |
| Anastomotic Leakage | 0.638 (0.096-4.197) | 1.000 |  | 1.709 (0.983-2.973) | 0.055 |
| Chylous Ascites | 3.286 (0.371-34.083) | 0.609 |  | 0.948 (0.501-1.796) | 0.871 |
| Pneumonia | 0.686 (0.205-2.295) | 0.540 |  | 1.337 (0.919-1.945) | 0.129 |
| Postoperative Length of Stay | - | 0.303 |  | - | 0.161 |

^*^ Anastomotic bleeding did not occur in transfused patients.

Table S6 Association between varying degrees of anemia and Clavien-Dindo classification

| Comparison groups | Total number of patients (case/control) | Number of patients without complications (case/control) | Clavien-Dindo classification (I/II/III/IV/V, case vs control) | Mean rank (case/ control) | Z-value | *P*-value ^a^ |
| --- | --- | --- | --- | --- | --- | --- |
| "Mild anemia + moderate anemia" vs "No anemia" | 609/609 | 298/300 | (89/190/24/7/1) vs (95/175/29/9/1) | 611/608 | -0.128 | 0.898 |
| "Moderate anemia" vs "Mild anemia + no anemia" | 97/231 | 44/111 | (11/36/3/2/1) vs (36/59/20/4/1) | 168/163 | -0.474 | 0.635 |
| "Moderate anemia" vs "No anemia" | 74/92 | 36/40 | (12/19/5/2) vs  (12/35/2/3) | 81/86 | -0.665 | 0.506 |

Data are presented as n.
^a^ Comparison of mean CDC grades between two groups using rank sum test

Table S7 STROBE statement checklist

|  | Item No | Recommendation |
| --- | --- | --- |
| Title and abstract | 1 | (a) Indicate the study’s design with a commonly used term in the title or the abstract  Retrospective cohort study as stated in the Abstract on page 1. |
|  |  | (*b*) Provide in the abstract an informative and balanced summary of what was done and what was found  Provided in Abstract on page 1 and 2. |
| Introduction | | |
| Background/rationale | 2 | Explain the scientific background and rationale for the investigation being reported  Included in the Introduction on pages 3 and 4. |
| Objectives | 3 | State specific objectives, including any prespecified hypotheses  Included in the Introduction on page 4. |
| Methods | | |
| Study design | 4 | Present key elements of study design early in the paper  Included in the Materials and Methods on page 5. |
| Setting | 5 | Describe the setting, locations, and relevant dates, including periods of recruitment, exposure, follow-up, and data collection  Included in the Materials and Methods on page 5, 6 and 7. |
| Participants | 6 | (*a*) *Cohort study*—Give the eligibility criteria, and the sources and methods of selection of participants. Describe methods of follow-up  Included in the Materials and Methods on page 5 and 6.  *Case-control study*—Give the eligibility criteria, and the sources and methods of case ascertainment and control selection. Give the rationale for the choice of cases and controls  *Cross-sectional study*—Give the eligibility criteria, and the sources and methods of selection of participants |
|  |  | (*b*) *Cohort study*—For matched studies, give matching criteria and number of exposed and unexposed  Included in the Materials and Methods on page 8.  *Case-control study*—For matched studies, give matching criteria and the number of controls per case |
| Variables | 7 | Clearly define all outcomes, exposures, predictors, potential confounders, and effect modifiers. Give diagnostic criteria, if applicable  Included in the Materials and Methods on page 6 and 7. |
| Data sources/ measurement | 8* | For each variable of interest, give sources of data and details of methods of assessment (measurement). Describe comparability of assessment methods if there is more than one group  Included in the Materials and Methods on page 7 and 8. |
| Bias | 9 | Describe any efforts to address potential sources of bias  Included in the Materials and Methods on page 7 and 8. |
| Study size | 10 | Explain how the study size was arrived at  Included in the Materials and Methods on page 7 and 8. |
| Quantitative variables | 11 | Explain how quantitative variables were handled in the analyses. If applicable, describe which groupings were chosen and why  Included in the Materials and Methods on page 9. According to this study, quantitative variables were divided into preoperative anemia group and non-anemia group. |
| Statistical methods | 12 | (*a*) Describe all statistical methods, including those used to control for confounding  Included in the Materials and Methods on page 8. |
|  |  | (*b*) Describe any methods used to examine subgroups and interactions  Not applicable. |
|  |  | (*c*) Explain how missing data were addressed  Included in the Materials and Methods on page 6. |
|  |  | (*d*) *Cohort study*—If applicable, explain how loss to follow-up was addressed  Not applicable.  *Case-control study*—If applicable, explain how matching of cases and controls was addressed  *Cross-sectional study*—If applicable, describe analytical methods taking account of sampling strategy |
|  |  | (*e*) Describe any sensitivity analyses  Included in the Materials and Methods on page 8. |

| Results | | |
| --- | --- | --- |
| Participants | 13* | (a) Report numbers of individuals at each stage of study—eg numbers potentially eligible, examined for eligibility, confirmed eligible, included in the study, completing follow-up, and analysed  Included in the Results on pages 10. |
|  |  | (b) Give reasons for non-participation at each stage  Not applicable. |
|  |  | (c) Consider use of a flow diagram  Included in Figure 1. |
| Descriptive data | 14* | (a) Give characteristics of study participants (eg demographic, clinical, social) and information on exposures and potential confounders  Included in the Results on pages 10 and 11. |
|  |  | (b) Indicate number of participants with missing data for each variable of interest  Included in Figure 1. |
|  |  | (c) *Cohort study*—Summarise follow-up time (eg, average and total amount)  As a retrospective study, aiming to collect patients’ complications during hospitalization. |
| Outcome data | 15* | *Cohort study*—Report numbers of outcome events or summary measures over time  Included in the Results on pages 13 and 14. |
|  |  | *Case-control study—*Report numbers in each exposure category, or summary measures of exposure |
|  |  | *Cross-sectional study—*Report numbers of outcome events or summary measures |
| Main results | 16 | (*a*) Give unadjusted estimates and, if applicable, confounder-adjusted estimates and their precision (eg, 95% confidence interval). Make clear which confounders were adjusted for and why they were included  Included in the Results on pages 11, and summarized in Tables 4 and additional file 1 Table S2. |
|  |  | (*b*) Report category boundaries when continuous variables were categorized  Not applicable. |
|  |  | (*c*) If relevant, consider translating estimates of relative risk into absolute risk for a meaningful time period  Not applicable. |
| Other analyses | 17 | Report other analyses done—eg analyses of subgroups and interactions, and sensitivity analyses  Included in the Results on pages 15, 16, 17 and summarized in Tables 4, 5 and S5. |
| Discussion | | |
| Key results | 18 | Summarise key results with reference to study objectives  Included in the Discussion on page 18. |
| Limitations | 19 | Discuss limitations of the study, taking into account sources of potential bias or imprecision. Discuss both direction and magnitude of any potential bias  Included in the Discussion on pages 21. |
| Interpretation | 20 | Give a cautious overall interpretation of results considering objectives, limitations, multiplicity of analyses, results from similar studies, and other relevant evidence  Included in the Discussion on pages 18. |
| Generalisability | 21 | Discuss the generalisability (external validity) of the study results  Included in the Discussion on page 21. |
| Other information | | |
| Funding | 22 | Give the source of funding and the role of the funders for the present study and, if applicable, for the original study on which the present article is based  Provided in the Funding section in Declaration in page 22 and 23. |
